# Wilms’ tumor 1-associating protein plays an aggressive role in diffuse large B-cell lymphoma and forms a complex with BCL6 via Hsp90

**DOI:** 10.1186/s12964-018-0258-6

**Published:** 2018-08-24

**Authors:** Yue Kuai, Xin Gong, Liya Ding, Fang Li, Lizhen Lei, Yuqi Gong, Qingmeng Liu, Huajiao Tan, Xinxia Zhang, Dongyu Liu, Guoping Ren, Hongyang Pan, Yaoyao Shi, Friederike Berberich-Siebelt, Zhengrong Mao, Ren Zhou

**Affiliations:** 1 Department of Pathology and Pathophysiology, Institute of Pathology and Forensic Medicine, Zhejiang University School of Medicine, Hangzhou, China; 2 Department of Medical Oncology, Institute of Clinical Science, Sir Run Run Shaw Hospital, Zhejiang University School of Medicine, Hangzhou, China; 3 Department of Pathology, the Second Hospital of Shaoxing, Shaoxing, China; 4 Epitomics (Hangzhou) Inc, Hangzhou, China; 5 Department of Orthopedics, the Second Affiliated Hospital, Zhejiang University School of Medicine, Hangzhou, China; 6 Department of Pathology, the First Affiliated Hospital, Zhejiang University School of Medicine, Hangzhou, China; 7 Department of Pathology, Sir Run Run Show Affiliated Hospital, Zhejiang University School of Medicine, Hangzhou, China; 8 Institute of Pathology, University of Würzburg, 97080 Würzburg, Germany

**Keywords:** WTAP, BCL6, Hsp90, Complex, DLBCL

## Abstract

**Background:**

Wilms’ tumor 1-associating protein (WTAP) is a nuclear protein, which is ubiquitously expressed in many tissues. Furthermore, in various types of malignancies WTAP is overexpressed and plays a role as an oncogene. The function of WTAP in diffuse large B-cell lymphoma (DLBCL), however, remains unclear.

**Methods:**

Immunohistochemistry was applied to evaluate the levels of WTAP expression in DLBCL tissues and normal lymphoid tissues. Overexpression and knock-down of *WTAP* in DLBCL cell lines, verified on mRNA and protein level served to analyze cell proliferation and apoptosis in DLBCL cell lines by flow cytometry. Finally, co-immunoprecipitation (Co-IP), IP, and GST-pull down assessed the interaction of WTAP with Heat shock protein 90 (Hsp90) and B-cell lymphoma 6 (BCL6) as well as determined the extend of its ubiquitinylation.

**Results:**

WTAP protein levels were consistently upregulated in DLBCL tissues. WTAP promoted DLBCL cell proliferation and improved the ability to confront apoptosis, while knockdown of *WTAP* in DLBCL cell lines allowed a significant higher apoptosis rate after treatment with Etoposide, an anti-tumor drug. The stable expression of WTAP was depended on Hsp90. In line, we demonstrated that WTAP could form a complex with BCL6 via Hsp90 in vivo and in vitro*.*

**Conclusion:**

WTAP is highly expressed in DLBCL, promoting growth and anti-apoptosis in DLBCL cell lines. WTAP is a client protein of Hsp90 and can appear in a complex with BCL6 and Hsp90 in DLBCL. Down-regulation of WTAP could improve the chemotherapeutic treatments in DLBCL.

**Electronic supplementary material:**

The online version of this article (10.1186/s12964-018-0258-6) contains supplementary material, which is available to authorized users.

## Background

Wilms’ tumor 1-associating protein (WTAP) is a nuclear protein first identified as a binding protein of Wilms’ tumor 1 (WT1) both in vitro and in vivo [1]. The importance of WTAP has been verified by knockout experiments, as WTAP-null mice die at embryonic day 6.5 [2] and WTAP heterozygous mice die at embryonic day 10.5 [3]. The biological function of WTAP in normal cells has been partly explored. Dynamic expression of WTAP and WT1 in smooth muscle cells (SMCs) can regulate cell proliferation [4]. WTAP expression can be enhanced by insulin-like growth factor-1 (IGF-1) conferring anti-apoptotic properties on smooth muscle cells [5]. Furthermore, in human umbilical vein endothelial cells, WTAP facilitates cell cycle progression by stabilizing cyclin A2 mRNA through an effect on its 3’UTR [2]. Meanwhile, accumulating evidences have shown that WTAP may play an aggressive role in some malignant tumors. For instance, WTAP is overexpressed in glioblastoma and regulates motility of glioblastoma cells by controlling the activity of EGFR [6]. In cholangiocarcinoma cells, WTAP regulates migration and invasion, where microarray studies showed that WTAP could induce the expression of MMP7, MMP28, cathepsin H and Muc1 [7]. Moreover, WTAP is a novel oncogene in acute myelogenous leukemia (AML) and a client protein of Heat shock protein 90 (Hsp90) [8]. Client proteins are drawn into a multi-protein complex by Hsp90, a molecular chaperone assisting in folding and stabilization of proteins [9]. Without the company of Hsp90, client proteins may be misfolded, subsequently ubiquitylated and proteasomally degraded [10]. Hence, Hsp90 is a rational therapeutic target for cancer [11].

B-cell lymphoma 6 (BCL6) is known client protein of Hsp90, i.e. *BCL6* might function as a stress response gene that forms part of a larger, post-transcriptionally regulated program governed by Hsp90 [12]. In the pathogenesis of Diffuse large B-cell lymphoma (DLBCL), BCL6 transcriptional repressor is the most frequently involved oncoprotein, which is required to sustain proliferation and survival of DLBCL cells through regulation of specific targets such as *PRDM1*, *c-Myc* or *PAX-5* [13]. However, relatively little is known about the contribution of WTAP – one further client protein of Hsp90.

DLBCLs are the most common B-cell non-Hodgkin lymphoma (NHL) in the world, comprising about 30–35% of all NHLs [14], which exhibit a heterogeneity in morphology, immunophenotype, genetics, and biological behavior [15]. Activated B-cell (ABC) and germinal-center B-cell (GCB) subgrous of DLBCL have been defined by gene-expression profiling, leaving approximately 10 to 20% of cases “unclassified” [16, 17]. Up to one third of DLBCL cases have abnormalities of *BCL6* and ~ 20% of cases have translocations of *BCL2* [18]. Although there are some patients, who can be cured of DLBCL, a substantial fraction of them (∼40%) die of this disease [19], pointing to the growing need to explore more specific drugs.

There are some in vitro studies, which provide evidence for the interaction of Hsp90 with WTAP as well as Hsp90 with BCL6. Since in a recent study Hsp90 was found to be frequently expressed in DLBCLs [20], we hypothesized that WTAP expression in DLBCL could be regulated by Hsp90 activity. In such a case, Hsp90 inhibition would affect the maintenance of WTAP and the protein’s function contributed by WTAP. Moreover, we speculated that WTAP might form a complex with BCL6 via Hsp90. Indeed, we could demonstrate that WTAP is not only highly expressed in DLBCLs and detectable in a complex with Hsp90 and BCL6, but mediates proliferation, while counteracting apoptosis.

## Methods

### Cell culture

HEK293T cell line was obtained from the Type Culture Collection of the Chinese Academy of Sciences (Shanghai, China); Dr. Xin Jiang (China) kindly provided DLBCL cell lines OCI- Ly10, OCI-Ly19, SU-DHL2 and SU-DHL4. The HEK293T cells were maintained in DMEM supplemented with 10% FBS (Gibco). The DLBCL cell lines were maintained in IMDM with 10% FBS (Gibco). Cultures were maintained in a 5% CO_2_ humidified atmosphere at 37 °C.

### Construction of vector

The *WTAP* gene was PCR-amplified from HEK293T cDNA and ligated into the pLVX-Puro vector (Clontech Laboratories) and pcDNA3.1-his-myc-B vector (Invitrogen), named WTAP-pLVX-Puro and pcDNA 3.1-WTAP, respectively. The *WTAP*-encoding sequence was also inserted into the pGEX-4 T-3 (Amersham biosciences) plasmid, called GST-WTAP thereafter. The *BCL6* gene was PCR-amplified from HEK293T cDNA and ligated into the pcDNA3.1-his-myc-B (Invitrogen), named BCL6-His.

### WTAP gain and loss of function experiments

WTAP-overexpressed lentivirus was packaged by different recombinant plasmids along with helper plasmids (psPAX2 and pMD2.G) in HEK293T cells, and virus supernatants were collected at 48 h and 72 h post-transfection. After concentration, recombinant WTAP-pLVX-Puro virus or control (pLVX-Puro) virus were infected into OCI-Ly19 cells. Additionally, WTAP-knock-down lentiviral infectious supernatant was obtained from Ibsbio company (China). WTAP target sequence was “GGGCAACACAACCGAAGAT”, the control sequence was “TTCTCCGAACGTGTCACGT”. WTAP-specific lentivirus was infected into OCI-Ly10 cells for knock-down, and control cells were generated using a non-target scramble. After infection, stable clones were selected with puromycin (Invitrogen) at a final concentration of 2 μg/ml.

### Immunohistochemistry

A total of 30 DLBCLs paraffin-embedded samples and 30 normal lymphoid tissues were obtained from the Second hospital of Shaoxing and the First Affiliated Hospital of Zhejiang University, respectively, after the necessary informed consent of patients. In each case, diagnosis of DLBCL was made according to the World Health Organization Classification of Tumors of Hematopoietic and Lymphoid Tissues [21]. This study was approved by the Ethics Committee of Zhejiang University (Hangzhou, China). IHC staining was performed using the EnVision system (Maixin Biotech) with antibodies for WTAP (1:1200, Abcam, ab155655). Data were calculated as previously described [22].

### RNA isolation and quantitative real time PCR (qRT-PCR)

Total RNA was isolated from cell lines by Trizol (Invitrogen) according to the manufacturer’s instruction, and reverse-transcribed using a PrimeScript RT reagent kit (Takara). Real-time PCR was performed three times in triplicate with SYBR Premix Ex TaqTM (Takara), using the 480II Real-Time PCR System (Roche). WTAP-F: TGTGCTGTGTAAGGGCATTCGTACTCATGC, WTAP-R: ACTGGGCAAACTTGGCAGTCATAAACCCAC; GAPDH-F: ACCACAGTCCATGCCATCAC; GAPDH-R: TCCACCACCCTGTTGCTGTA. The reactions were incubated at 95 °C for 5 min, followed by 40 cycles of 95 °C for 30 s, 60 °C for 30 s and 72 °C for 30 s. The relative amount of WTAP mRNA was normalized to that of GAPDH. Relative expression levels were normalized to GAPDH and calculated with the 2-ΔΔct method. Each experiment was performed independently at least three times.

### Protein isolation and western blotting

Nuclear and cytoplasmic proteins were obtained according to the manufacturer’s instruction (Beibokit, China). The total protein samples were harvested with 1 × SDS loading buffer or RIPA and then resolved by SDS- polyacrylamide gel electrophoresis (PAGE), electrophoretically transferred to PVDF membranes (Millipore) and blocked in nonfat milk in TBS/Tween-20. Antibodies were specific for BCL6 (N3) (Santa cruz, sc-858), WTAP (Abcam, ab195380), anti-Hsp90 (Abcam, ab203126). The anti-GAPDH (Cell Signaling Technology, 5174), anti-HistoneH2A (Cell Signaling Technology, 7631), anti-LaminA/C (Abcam, ab108595), anti-ubiquitin, Lys48-specific Clone Apu2 (Millipore, 2145090), anti-His and anti-GST antibodies were purchased from CWBiotech. The experiments for the immunoblotting were performed at least three times.

### Proliferation assay

Cells (1 × 10^4^/well) were seeded into 96-well plates in triplicates, and were cultured in 100 μL IMDM containing 10% FBS for 3 days. The Cell Counting Kit-8 (CCK8) was used to evaluate cell proliferation. Briefly, 10 μL CCK8 solution was added to each plate, and cells were incubated for 4 h at 37 °C. Cell viability was measured at 450 nm by SpectraMax M5.

### Apoptosis assay

OCI-Ly19 cells were infected with control or WTAP- pLVX-Puro lentivirus and then cultured for 48 h; an optimum concentration of puromycin was used until the cells in a blank group died. We cultured cells with hydrogen peroxide (300 mM) combined with serum deprivation for 16 h. 2 × 105 cells were harvested with ice-cold phosphate buffered saline (PBS). SU-DHL2 and SU-DHL4 were cultured in PU-H71 (1.5 μM) for 48 h, and then added Etoposide (10 µM) for another 24 h. Cells were also collected and stained with Annexin V-PE along with 7-AAD to detect apoptosis by flow cytometry. WTAP-knocked down OCI-Ly10 cells or the control cells were cultured in the presence of Etoposide or DMSO for 24 h. The final concentration of Etoposide was 10 μM. Cells were also collected and stained with Annexin V-PE along with 7-AAD. The quantitative analysis of the percentage of positive cells was reported from three independent experiments.

### Immunofluorescence staining

After cultures with PU-H71 or DMSO for 48 h, OCI-Ly10 cells were washed twice in PBS, spread onto slides, air dried and fixed in acetone at − 20 °C for 15 min. Immunofluorescence staining was performed as described previously [13]. After treatment of 0.3% Triton X-100 (KeyGen Biotech) for 10 min, the slides were blocked with Goat serum (Boster Biological Technology) for 1 h at room temperature. The cells were then incubated with the primary antibody against WTAP (diluted 1:50, Santa Cruz, sc-374280) and BCL6 (diluted 1:100, Abcam, ab33901) overnight at 4 °C. Signals were detected with Alexa Fluor 488 and Alexa Fluor 594, respectively. Nuclei in live cells were stained with 4', 6-diamidino-2-phenylindole (DAPI), and visualized with laser confocal microscopy (NikonA1R).

### Co-immunoprecipitation (Co-IP)

HEK293T cells were co-transfected with BCL6-His and pcDNA3.1-WTAP vectors by LipofectamineTM 3000 (Invitrogen). 48 h after transfection, collected cells were lysed on ice for 1 h using RIPA buffer (Beyotime) with 1 mM Phenylmethanesulfonyl fluoride. Co-IP was performed as described previously [13]. Briefly, 1 μl anti-WTAP antibody (Abcam, ab195380) was added to the whole-cell lysate. The immune complexes were incubated at 4 °C with rotation and collected using 20 μl protein G Plus-Agarose Immuno-precipitation Reagent (Santa Cruz). After that, the collected agarose beads were boiled in SDS sample buffer and analyzed by western blotting. LaminA/C is a nuclear protein and served as a negative control. All the experiments were performed in triplicates.

### Immunoprecipitation

OCI-Ly19 were harvested from a six-well plate, the cells were lysed on ice for 1 h using RIPA buffer (Beyotime) with 1 mM Phenylmethanesulfonyl fluoride. 1 μl anti-WTAP antibody (Abcam, ab195380), 5 μl anti-BCL6 (N3) (Santa cruz, sc-858) and 1 μl IgG (Millipore) was added to the whole-cell lysates, respectively. The immune complexes were incubated at 4 °C with rotation for 6 h, then supplemented with 20 μl protein G Plus-Agarose Immuno-precipitation Reagent (Santa Cruz) and incubated at 4 °C for 12 h. After that, the collected agarose beads were boiled in SDS sample buffer and analyzed by western blotting. All the experiments were performed in triplicates.

### GST fusion proteins and his-tag-purified protein pull-down assay

A total of 100 μl GST fusion proteins of GST-WTAP or GST vector was purified and immobilized on GST-immunomagnetic beads (Beaverbio, China) to pull down the GST-bound form of His-tag fusion protein at 4 °C for 4 h. BCL6-His fusion protein was extracted from HEK293T cells transfected with BCL6-His and purified by His-immunomagnetic beads according to the manufacturer’s instructions (Beaverbio, China). at 4 °C for 5 min × 3 with a buffer containing 1 ml PBS and 1% TritonX-100, the bound proteins were eluted by boiling in 2 × loading buffer followed by SDS-PAGE and detected by western blotting analysis with anti-GST antibodies (CWBiotech, China) and anti-Hsp90 antibodies (Abcam, ab203126).

### GST fusion proteins and cell lysates pull-down assay

A total of 100 μl of GST fusion proteins of GST-WTAP or GST was purified and immobilized on GST-immunomagnetic beads (Beaverbio, China) to pull down the GST-bound form of proteins extracted from the HEK293T cells transfected with BCL6-His.The following progress was the same as above described.

### Statistical analysis

Differences between the expression score of immunohistochemistry in lymphoid tissues and DLBCLs were assessed using the Student’s t-test. For in vitro and in vivo assays, data were analyzed using Student’s t-test or one-way ANOVA to compare the results among experimental groups and control groups. Statistical analysis was carried out using Graph Pad Prism version 5. Significance level was set at *P* < 0.05.

## Results

### WTAP was overexpressed in DLBCLs

**Fig. 1:**
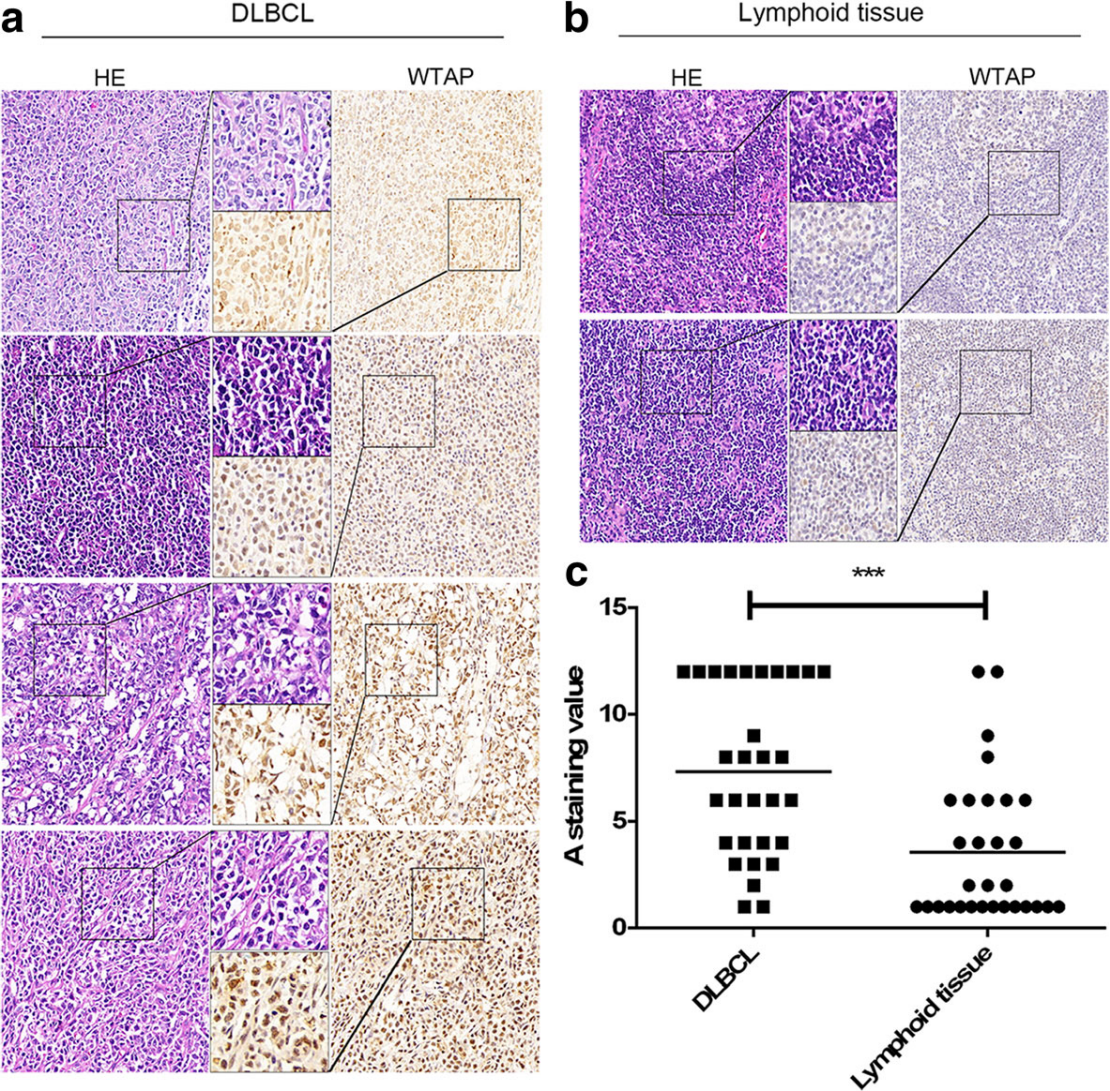
WTAP is overexpressed in DLBCL. **a** Representative H&E-stained, WTAP (brown) -stained sections of DLBCL tissues; Different grades in the intensity of WTAP staining are presented. **b** H&E-stained and WTAP-stained sections of lymphoid tissues. **c** States of WTAP protein expression in patients (*n* = 30) is significantly higher than in normal lymphoid tissues (*n* = 30) (***P < 0.001, Student’s t-test). These sections were evaluated by two pathologists (double blinded). A staining index (values 0–12) were obtained for the intensity of WTAP-positive staining (negative, 0; weak 1; moderate, 2; or strong, 3 scores) and the proportion of immunopositive cells (< 25%, 1; 25–50%, 2; 50–75%, 3; > 75%, 4 scores) was calculated

To determine the expression status of WTAP in DLBCLs, we performed immunohistochemistry staining using DLBCL patients’ tissues (*n* = 30) (Additional file 1: Table S1) and normal lymphoid tissues (*n* = 30). Indeed, stainings revealed nuclear expression of WTAP (Fig. 1a). Importantly, WTAP protein was widely overexpressed in the cancerous areas of DLBCL tissues and, compared with the control group (Fig.1b), a higher level of WTAP was observed in DLBCLs tissues (Fig. 1c). Many, but not all DLBCL tissues overexpressed it compared with normal lymphoid tissues. The score of each sample was calculated as previously described [22].

### Roles of WTAP in proliferation and apoptosis of DLBCL cells

**Fig. 2:**
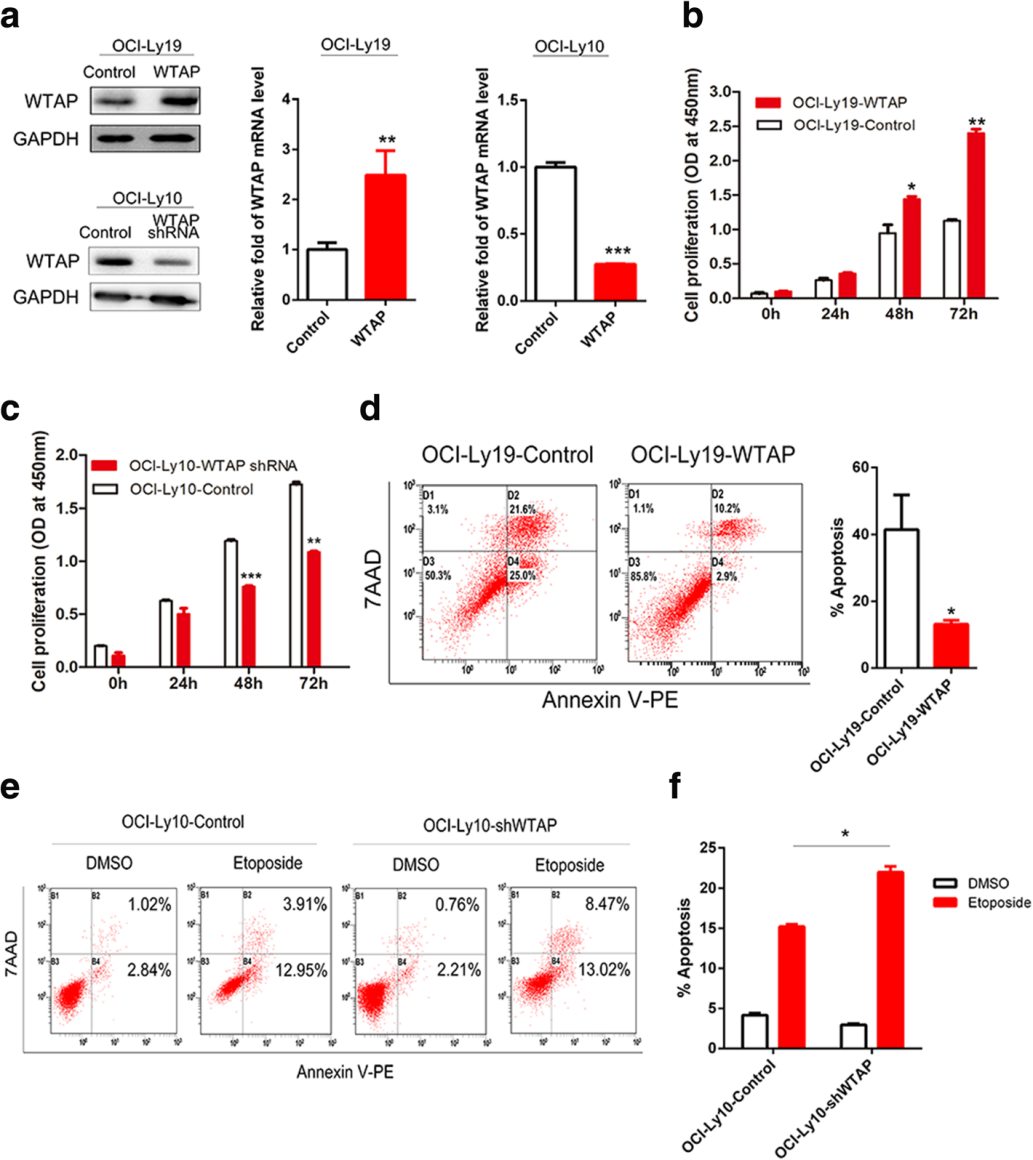
WTAP supports proliferation and counteracts apoptosis of DLBCL cells. **a** OCI-Ly19 and OCI-Ly10 cells, 48 h post-infection. Expression of WTAP was checked on the level of protein and mRNA by using western blotting and qPCR. Mean ± s.d. of three technical replicates were plotted. **P < 0.01, ***P < 0.001. **b**, **c** We infected OCI-Ly19 and OCI-Ly10 by WTAP-overexpressing lentivirus or WTAP-knocked down lentivirus. Then cells were cultured in 96 cell panels for 0, 24, 48, and 72 h. Cell viability was measured by CCK8 assay.*, *P* < 0.05, **, *P* < 0.01 and ***, *P* < 0.001 compared with control group. **d** OCI-Ly19-WTAP and the control group, OCI-Ly19-control, were cultured in serum deprivation medium and treated with hydrogen peroxide (300 mM) for 16 h. The apoptosis assay was detected by flow cytometry. *P < 0.05 compared with the control group. **e** OCI-Ly10-shWTAP and OCI-Ly10-control cells were cultured in medium with Etoposide (10 μM) or DMSO for 24 h. **f** The apoptosis was assayed by flow cytometry. *, *P* < 0.05 compared with the control. The quantitative analysis of the percentage of positive cells was reported from three independent experiments

To examine roles of WTAP in DLBCL, we overexpressed or knocked-down WTAP using lentivirus. We checked the efficiency of infection by western blotting and real-time PCR (Fig. 2a). Two days after infection with overexpressing or knocking-down lentivirus, an optimum concentration of puromycin was used until cells in a blank group died. Proliferation assays were carried out with the selected cells. WTAP positively influenced cell proliferation, as the proliferation of OCI-Ly19 cells, which were infected with WTAP overexpressed lentivirus, was higher than the control group (Fig. 2b). On the reverse, when WTAP had been knocked-down, the proliferation of OCI-Ly10 cells was inhibited (Fig. 2c). To determine the role of WTAP during the process of DLBCL cell apoptosis, we cultured WTAP overexpressing cells with hydrogen peroxide combined with serum deprivation for 16 h and then we checked the apoptosis level with flow cytometry. A significant lower apoptosis rate was revealed in the group overexpressing exogenous WTAP (Fig. 2d). In knocked-down cells, there was no obvious difference between the two groups (data not shown). However, when we treated WTAP-knock-down cells and control cells with Etoposide or DMSO, respectively, a distinctive higher apoptosis rate was evident in the WTAP-reduced group upon Etoposide treatment (Fig. 2e, f). Together, WTAP supports proliferation and counteracts Etopside-mediated apoptosis induction. Hence, we speculate that a DLBCL patient, who has a higher level of WTAP expression, may experience a poor therapeutic efficiency of chemotherapy.

### Hsp90 can stabilize WTAP on the protein level

**Fig. 3:**
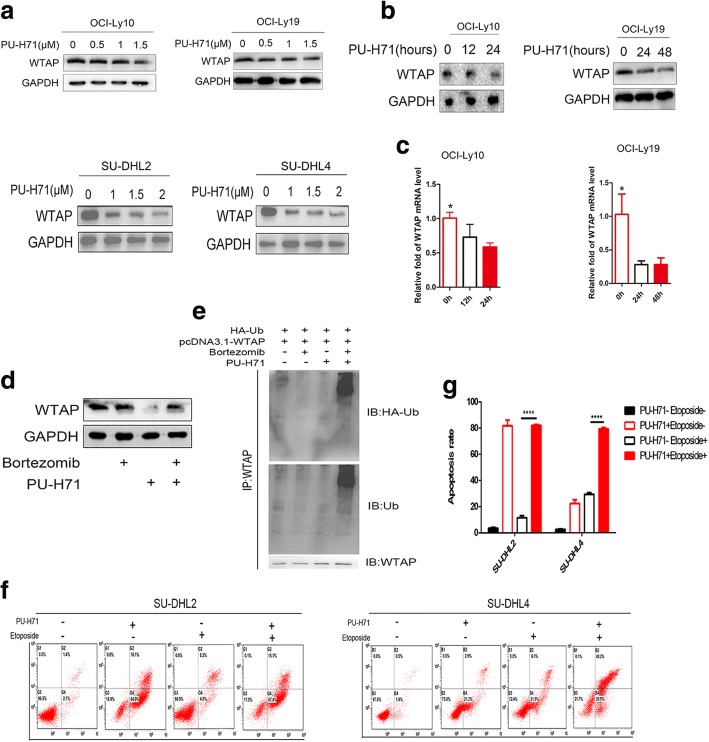
Hsp90 can stabilize WTAP on the level of protein and mRNA. **a** OCI-Ly10, OCI-Ly19, SU-DHL2 and SU-DHL4 were cultured with different PU-H71 concentrations for 24 h. WTAP protein level was detected by western blot. **b** Cells treated with PU-H71 (1 μM) for various times, protein expression level were assessed by western blotting. **c** mRNA levels of *WTAP* were checked by qRT-PCR. Results are expressed as fold difference compared to baseline (time 0 h) and were normalized to GAPDH. Experiments were performed three times, each with duplicate qPCR measurements. Error bars represent the s.e.m. from three replicates. **d** Cells were pretreated with 100 nM Bortezomib, an inhibitor for 26 s proteasome, or DMSO only for 2 h. Then cells were cultured in the presence of 1 μM PU-H71 or DMSO for 6 h. WTAP expression was checked by western blotting. **e** HEK293T cells were transfected with pcDNA3.1-WTAP and HA-Ub, then treated with PU-H71 or DMSO for 24 h. Cell were collected to add anti-WTAP, and ubiquitination level were check. **f**, **g** Cells were pretreated with 1.5 μM PU-H71 or DMSO for 24 h, then cultured with Etoposide (10 μM) for another 24 h. The apoptosis rates were detected by flow cytometry. These experiments were repeated three times

In a recent paper, H Bansal et al. have proven that in vitro Hsp90 could interact with WTAP, whereas in AML cell lines the expression of WTAP could be affected by inhibitors of Hsp90 [8]. Here, we were wondering about the relationship between WTAP and Hsp90 in DLBCL cell lines. Firstly, we chose the cell lines OCI-Ly10 and SU-DHL2, resembling the ABC types as well as OCI-Ly19 and SU-DHL4 which are GCB types as experimental subjects. After being cultured in the presence of PU-H71, an Hsp90 inhibitor, the level of expression of WTAP was changing with concentration and time (Fig. 3a, b). Meanwhile, WTAP is described to associate with the METTL3/14 heterodimer and to influence RNA processing including RNA stability [23]. Interestingly, we show here that reduced WTAP protein levels provoke less WTAP RNA (Fig. 3c). Since 3 AU-rich elements (ARE) could be detected with the help of an ARE database (data not shown), it is tempting to speculate that WTAP stabilizes its own RNA via these RNA-destabilizing motifs.

Lack of Hsp90 interaction might expose WTAP to ubiquination and proteasomal degradation [8]. Hence, we pretreated cells with Bortezomib, a 26 s proteasome inhibitor or DMSO for 2 h, and subsequently cultured them with PU-H71 for 6 h. We found that Bortezomib could prevent the actions of PU-H71 (Fig. 3d). In line, higher levels of ubiquitinated WTAP were observed in the presence of the combination of Bortezomib and PU-H71 compared with either agent alone (Fig. 3e), revealing that PU-H71-mediated degradation was depend on ubiquitin-proteasome pathway. Then we wondered if PU-H71, i.e. Hsp90 inhibition, impacts on apoptosis as well. We pretreated SU-DHL2 and SU-DHL4 cells with PU-H71 or DMSO for 24 h and then cultured them with Etoposide for another 24 h. Indeed, groups pretreated with PU-H71 demonstrated significantly higher levels of apoptosis (Fig.3f). In sum, similar to other Hsp90 clients, WTAP expression levels seem to be primarily regulated via protein stability.

### WTAP forms a complex with BCL6 via Hsp90 in vivo and in vitro

**Fig. 4:**
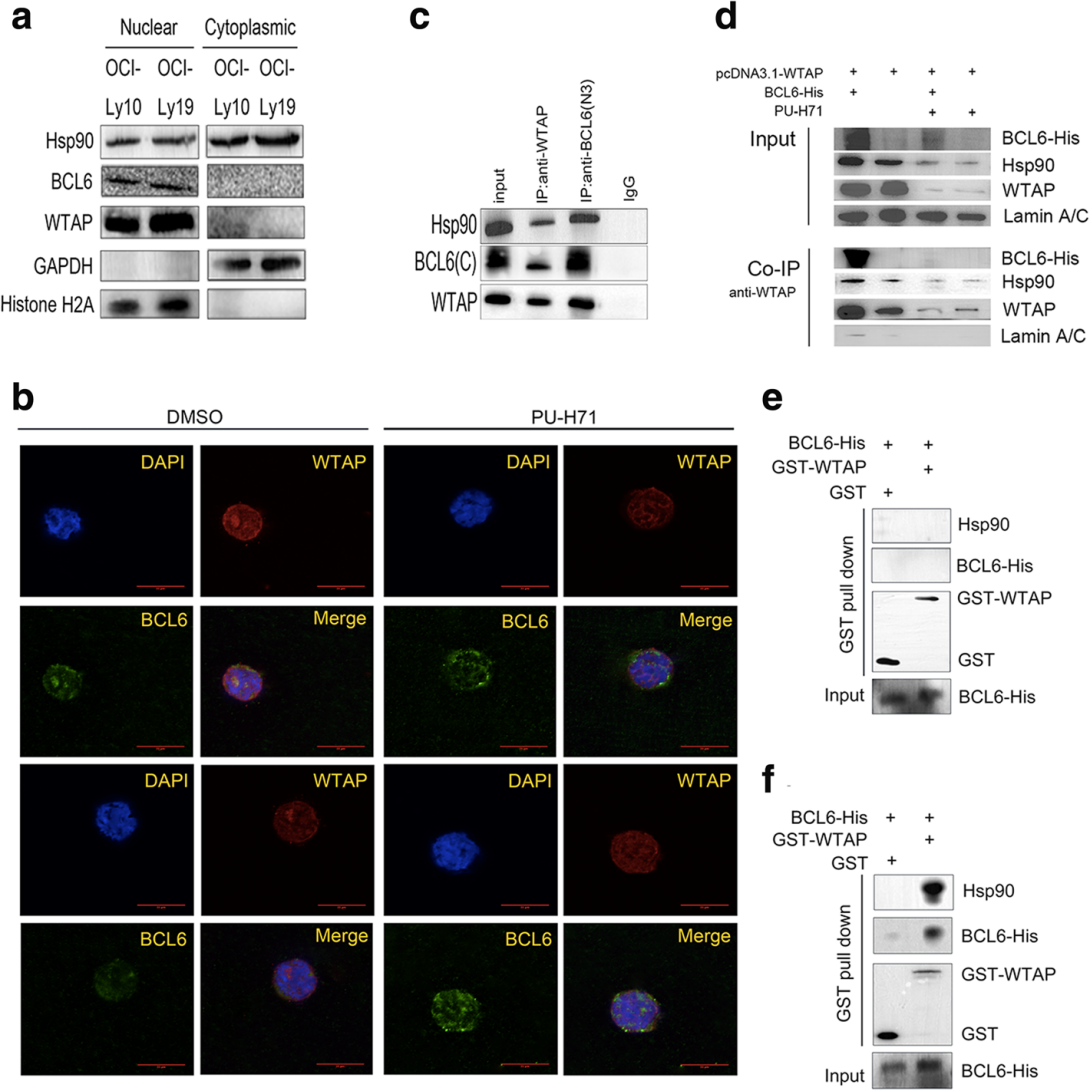
WTAP forms a complex with BCL6 via Hsp90. **a** OCI-Ly10 and OCI-Ly19 nuclear and cytoplasmic extracts were detected by Western blotting. **b** Co-localization of endogenous WTAP (red) and BCL6 (green) in DLBCL cells was detected by antibody stainings and confocal microscopy. When indicated, cells had been treated with PU-H71 (1 μM). Co-localization is shown by merge (yellow or orange). Nuclei were counterstained with DAPI (blue). **c** OCI-Ly19 cell lysates were supplemented with anti-WTAP, anti-BCL6 and negative control IgG antibodies. The protein complex was assessed against indicated antibodies. **d** Exogenous expression of WTAP and BCL6 was achieved by expression plasmids (pcDNA3.1-WTAP and BCL6-His) transfected into HEK293T cells and cultured for 24 h. PU-H71 (1.5 μM) or DMSO was added for another 24 h. Whole cell lysates were precipitated by anti-WTAP antibodies. WTAP, BCL6 and Hsp90 protein level were detected by the respective antibodies. LaminA/C as a nuclear protein served as a negative control. **e**, **f** GST pull-down assay to determine if WTAP interacts with BCL6 via Hsp90, either with GST-WTAP and BCL6-His only (**e**) or in the presence of whole cell extracts (**f**)

BCL6 as a transcriptional repressor is the most involved oncoprotein in DLBCL and therefore evaluated as a target for therapy [24]. In about 40% DLBCLs, the constitutive expression of BCL6 is related to translocations and mutations of its promoter [25]. However, there are still many other DLBCLs expressing BCL6 without genetic alterations, suggesting that additional factors can also sustain BCL6 expression or interact with it for some biological function. Hsp90 maintains the stability of the BCL6 protein [12]. In this study, we gained indications that also WTAP could be stabilized upon an interaction with Hsp90. In line, when cells cultured in the presence of an Hsp90 inhibitor, the WTAP expression level was reduced, which is consist with a previous study [8]. Yet, Hsp90 is usually cytoplasmic, whereas BCL6 and WTAP are localized in the nucleus. In contrast to the normal setting, we demonstrate here that Hsp90 localized to both the cytoplasmic and nuclear fractions of DLBCL cell lines (Fig. 4a). We wondered, whether the two nuclearly expressed client proteins of Hsp90, WTAP and BCL6, were co-located in cells or had some physical relationship to each other. To address this, we preformed co-localization studies using immunofluorescence. Indeed, the stainings elicited that WTAP and BCL6 were co-located in DLBCL cells (Fig. 4b). This phenomenon disappeared when cells had been treated with the Hsp90 inhibitor PU-H71, suggesting that Hsp90 has an effect on the co-localization. To determine whether Hsp90, WTAP and BCL6 could form a complex in cells, without either protein being overexpressed, immunoprecipitation experiments were carried out using OCI-Ly19 cell lysates. The DLBCL cell line OCI-Ly19 is known to express BCL6 and additionally, as shown by western blotting, endogenous WTAP and Hsp90 (Fig. 4a). The immunoprecipitations were carried out and after subsequent western blotting analysis with anti-WTAP antibody, WTAP and Hsp90 could be detected not only in the Co-IPs carried out with the anti-WTAP antibody, but could also be discovered in the anti-BCL6 Co-IPs, thus indicating that WTAP, Hsp90 and BCL6 formed a complex in vivo (Fig. 4c). Similarly, we performed Co-IPs after transfection of BCL6-his and pcDNA3.1-WTAP expressing plasmids into HEK293T cells. In parallel, cell were additionally treated with PU-H71. Firstly, WTAP interacts not only with endogenous Hsp90, but also with exogenous BCL6 if provided (Fig. 4d). Secondly, PU-H71 treatment, i.e. Hsp90 inhibition, obstructed the complex formation (Fig. 4d). Consequently, WTAP must be assumed to interact with BCL6 directly or indirectly via the chaperone Hsp90. Now GST pull-down assay revealed the presence of proteins in vitro, but affinity-purified GST-WTAP and the His-tagged fusion protein BCL6-His, did not bind to each other directly (Fig. 4e). In contrast, when whole cell lysates – presumably containing Hsp90 – were provided during GST-WTAP pull-down assay, BCL6-His and Hsp90 could be pulled down by GST-WTAP, but not GST (Fig. 4f). From this, we conclude that WTAP forms a complex with BCL6 via Hsp90 in vivo and in vitro.

## Discussion

WTAP is a nuclear protein which was first regarded as a housekeeping protein because of its ubiquitous expression pattern [1]. However, with an increase in the number of studies, WTAP has predominantly been described in the context of proliferation, apoptosis, migration and invasion of tumor cells [6, 7]. Accordingly, in mammalian cells, WTAP forms a nuclear complex with METTL3 and METTL14, which methylates *N*6-adenosines of RNA involved in cell cycle regulation, post-transcriptional mRNA metabolism and splicing [26–29]. WTAP, as the name indicates, partners with the Wilms’ tumor 1 (WT1), which has an oncogenic potential in leukemogenesis [30]. In AML, WTAP itself was identified as an oncogene [8]. In the present study, we show that WTAP is overexpressed in DLBCL and that it supports proliferation and inhibits apoptosis of DLBCL cells.

In particular, overexpression of WTAP could up-regulate the proliferation in DLBCL cell lines and knocked-down of WTAP would suppress cell proliferation. Apoptosis assays showed that overexpressed WTAP reduces the apoptosis rate of cells. Per se, knockdown of WTAP had no significant influence on apoptosis. However, after treated with Etoposide, an anti-tumor drug, the apoptosis rate in this experimental group was obviously higher than in the untreated control group. These results strongly suggest that WTAP acts as an aggressor in DLBCL and that the patients who exhibit a higher level of WTAP expression might have a poor curative effect on chemotherapy. This prompted us to address the underlying mechanism of WTAP stable and enhanced expression in DLBCLs.

**Fig. 5:**
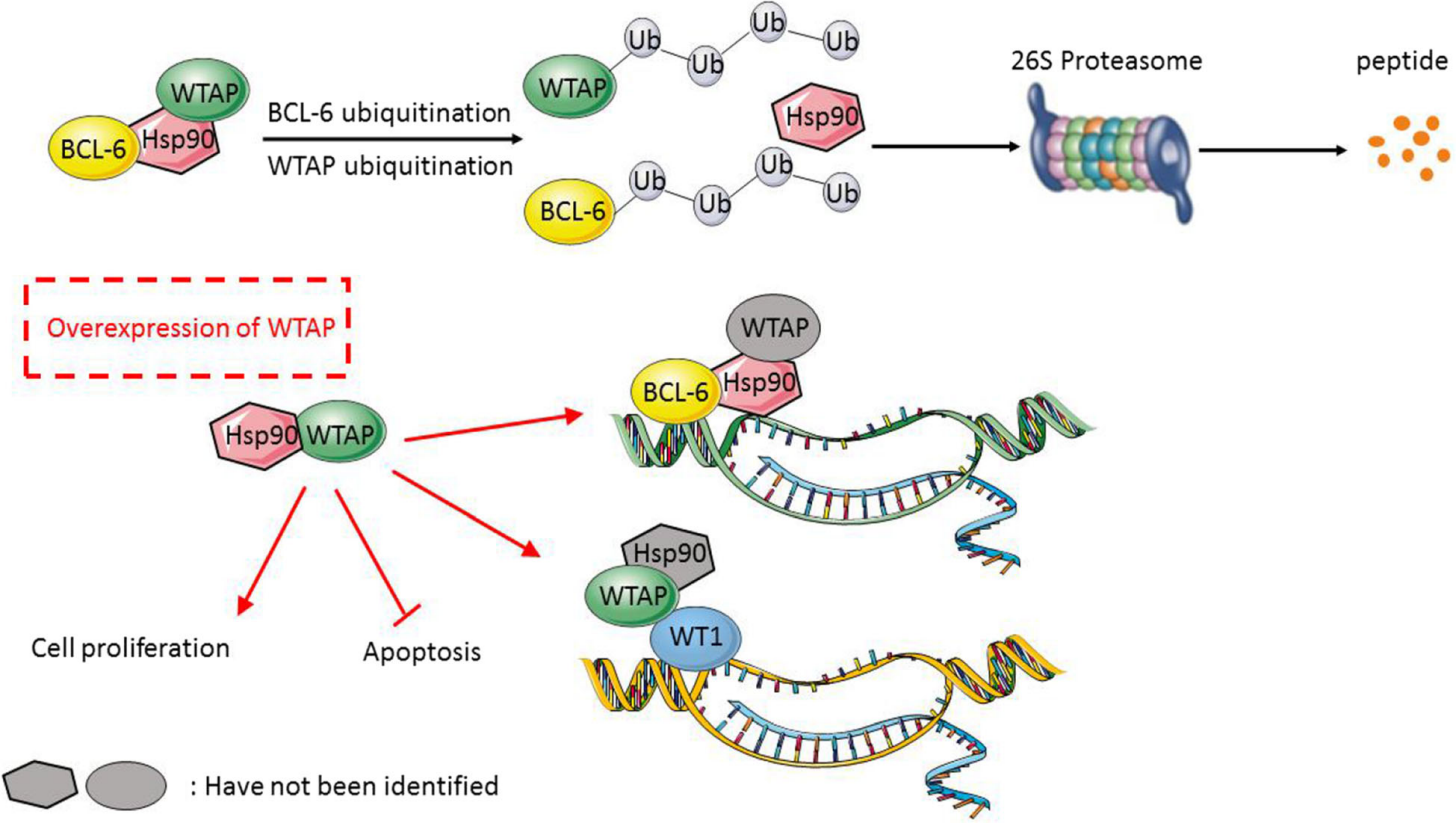
Proposed model of the interplay between Hsp90, WTAP and BCL6. Hsp90 could form a complex with WTAP and BCL6 to stabilize their expression on protein level. When WTAP and BCL6 separate from Hsp90, these two proteins would be degraded by the ubiquitin-proteasome pathway. The overexpressed WTAP in DLBCL cells and tissues could promote proliferation and reduce apoptosis. The described interaction of WTAP with WT1 could also involve Hsp90. The function of the WTAP, BCL6 and Hsp90 complex, besides stabilization, could lie in the process of transcriptional activity

DLBCL cells seem to be in a stressed state, because of the presence of mutant proteins and rapid proliferation, which puts additional pressure on controlling proteostasis [10]. In this stage, molecular chaperones are essential, as they ensure that changes that affect proteins are “buffered” to guarantee proteostasis and thus cellular homeostasis (Fig. 5). Thus, Hsp90 plays an important part in stabilization of client proteins. As we know, chaperone Hsp90 maintains the stability of many tumor-promoting oncoproteins [31]. Considering the elucidated oncogenic function of WTAP in DLBCLs, the complex with Hsp90 may indicate a target for DLBCL treatment. In case one is able to separate the client proteins from Hsp90, they will be degraded by the ubiquitin-proteasome pathway [31] (Fig. 5). Accordingly, we show in the present study that Hsp90 stabilizes WTAP as a protein. In addition, we determined that WTAP-Hsp90-BCL6 could form a protein complex in vivo and in vitro. Therefore, addressing Hsp90 by an inhibitor would leave its many client proteins – including BCL6 and WTAP – unprotected and prone to degradation.

Strikingly, *WTAP* mRNA was additionally reduced upon Hsp90 inhibition. Since WTAP – via its association with the METTL3/14 heterodimer – impacts on RNA stability [23], we hypothesize that in malignant DLBCL cells abundant WTAP protects *WTAP* mRNA from degradation, while this positive feedback loop can be interrupted by Hsp90-directed drugs and successive WTAP ubiquitination and proteasomal degradation. Interestingly, a similar scenario has been documented for BCL6, also bound to Hsp90 [12], degraded upon Hsp90 inhibition and subsequent less abundant RNA as well. One might speculate the here reported trimer of WTAP-Hsp90-BCL6 to stabilize all RNAs, which possess ARE elements recognized by WTAP-METTL3/14.

BCL6 was initially discovered as a transcriptional repressor in B-cell lymphomas, in which it drives the malignant phenotype by binding to hundreds of target genes, and then repressing these genes by recruiting several different chromatin modifying corepressor complexes to repress DNA damage checkpoints and block B-cell terminal differentiation [24]. Hsp90 could bind with BCL6 to perform as a transcriptional complex repressor, which has been verified by ChIP (Chromosome immunoprecipitation) in previous studies. *ATR, TP53* and *CD69* are target genes of BCL6. Their messenger RNA abundance can be derepressed by PU-H71 in a time-dependent manner. In other words, Hsp90 may thus function as a corepressor for BCL6 by maintaining it in a stable conformation directly within the gene repressing complexes [12] (Fig. 5). Indeed, as a transcriptional repressor, BCL6 inhibits numerous target genes by recruiting co-repressors (most potently in a ternary complex with BCOR and SMRT) and stabilized by Hsp90 at regulatory gene elements [12, 32, 33]. BCL6 also regulates B-cell responses to chemokines and cytokines [32, 34], cell cycle control, either directly or indirectly, by repressing target genes or interacting with Miz1 to represses transcription of CDKN1 [35, 36]. BCL6 could form a complex or directly function as a transcription factor or an oncology protein.

Whether the here described complex of WTAP-Hsp90-BCL-6 is involved in transcriptional activity and/or elicits other functions is still not known. Previous studies have shown WTAP could bind to Wilms’ tumor 1 (WT1), a transcription factor, forming a complex which affects transcriptional targets in smooth muscle cells (SMCs) [4]. Those targets have long been known to act as tumor suppressors in Wilms’ tumor in other neoplasms, including lung, kidney, and breast carcinoma, as well as in leukemia like acute myeloid leukemia (AML) and myelodysplastic syndrome [37, 38]. However, WT1 seems to play an opposite role to an oncogene. This may be due to a two-sided function of WT1 promoting differentiation of cells in the genitourinary tract, while maintaining an immature mesenchymal state in other tissues [39]. In line, the complex of WTAP-WT1 is involved in Wnt signaling in colon cancer development [40]. In DLBCL, WT1-positive patients showed significantly worse overall and disease-free survival under the same treatment protocols [41]. WTAP conforms complexes with – at least – two transcriptional factors, BCL6 and WT1, respectively. Whether Hsp90 is involved in both scenarios is unknown (Fig. 5). Maybe WTAP is primarily stabilized in the complex with Hsp90 and from this has the possibility to from alternate complexes with WT1 and METTL3/14 to exert its pro-tumorigenic potential. However, although the number of DLBCLs tissues examined in this study was not enough for generalization and more thorough studies in the future will clarify if high WTAP expression is a general feature of DLBCLs or describes a more aggressive subtype, we proclaim in any case to consider Hsp90 inhibition to be included during chemotherapy if WTAP expression is high.

## Additional file

Additional file 1: **Table S1.** The information of DLBCL samples. (DOC 30 kb)

## Abbreviations

ABC: Activated B cell
BCL6: B-cell Lymphoma 6
DLBCL: Diffuse large B-cell lymphomas
GAPDH: Glyceraldehyde-3-phosphate dehydrogenase
GCB: Germinal Center B cell
Hsp90: Heat shock protein 90
WTAP: Wilms’ tumor 1-associating protein
