# Supplementary material for: Wilms’ tumor 1-associating protein plays an aggressive role in diffuse large B-cell lymphoma and forms a complex with BCL6 via Hsp90
Source: Cell Commun Signal. 2018 Aug 24;16:50. doi: 10.1186/s12964-018-0258-6 (PMC6108153; doi:10.1186/s12964-018-0258-6)
Supplement: Supplementary file 1 — Table S1. The information of DLBCL samples. (DOC 30 kb) [file 12964_2018_258_MOESM1_ESM.doc]

| Table S1 |
| --- |

| Male 18  Female 12  Age(year) <40 1  40-59 11  60-79 17  >80 1  DLBCL-GCB 13 P=0.554  DLBCL-non-GCB 17  WTAP expression^#^  Low expression(≤ median) 10  High expression(> median) 20 |
| --- |
| ^#^WTAP expression were subdivided according to the median values of the study cohort into two categories: low expression (≤median) and high expression (>median)(13). |
